# Progress in Ophthalmology

**Published:** 1899-03-11

**Authors:** 


### Progress in Ophthalmology.

**"Protargol in Conjunctivitis."**—"Protargol" still seems to be the "hero of the hour" in the treatment of conjunctivitis, from the catarrhal right up to gonorrhœal, and even the Egyptian variety. 1. Dr. Darier[1] gives a most glowing account of its efficacy—he is even more sanguine than Dr. Chenery, whose account of protargol we mentioned in the last number of these notes. The present writer says: "The protargol solutions that I employed in treating purulent ophthalmia were a 50 per cent. solution for abortive treatment, after which a 25 per cent. solution should be applied for several days after the cessation of the discharge, also a 5 per cent. to 10 per cent. solution should be used by the patient as an eye-wash in the intervals of daily cauterisations by the stronger solutions. He furthermore continues, "From the day I first applied this treatment I have attended 320 patients suffering from ophthalmia, and all have been rapidly cured by protargol—one had never seen such a large number of children attacked by acute ophthalmia cured in less than five days. If the patient presents himself for treatment at the commencement of the attack—say the first or second day—and a single cauterisation with a 50 per cent. solution of protargol be made, followed by the instillation of a 5 to 10 per cent. collyrium of protargol every two or three hours, one frequently finds that the patient reappears on the following day perfectly cured, although it is advisable he should continue with the collyrium for several days further. If the suppuration has established itself, and the discharge becomes very abundant, one or two cauterisations a day, with instillation of the collyrium in the intervals should be made, after which a cure will be effected in two, three, or eight days at the most, but in these cases one must always continue the cauterisation with a 25 per cent. solution for several days to avoid a recurrence."

He continues: "Patients generally bear protargo solutions well, and it is only rarely, even after the application of a 50 per cent. solution, that a pseudo-membranous exudation is seen on the conjunctiva; in such a case the only thing necessary is to evert the lids some little time after the cauterisation and remove the false membrane with a tampon of cotton-wool, wash the surface gently with boric acid solution, and drop in the 5 per cent. collyrium, after which the patient experiences great relief." To this description we may add the testimony of Pisenti,[2] of Perugia, who says he has tried the above preparation of silver for the last six months in various types of ophthalmia and inflammation of the lachrymal canal. He appears to use much milder strengths than Darier, as he says protargol, even in 10 per cent. solution (strongest he has used), does not cause so much or such lasting pain as a 2 per cent. solution of nitrate of silver. In ophthalmia neonatorum he recommends a 10 per cent. solution two or three times a day. For catarrhal ophthalmia 2 per cent. to 5 per cent. is used. All seem to have noticed that protargol has no very great effect on granular conjunctivitis. Protargol, according to Pisenti, does not cause any of those changes in the corneal epithelium which may be sometimes seen after the application of nitrate of silver, but it has nevertheless a penetrating power five times greater. It is also an active germicide where the gonococcus is present. Valençon[3] divides the common inflammations of the conjunctiva into three varieties, viz.: (1) Acute contagious conjunctivitis, due to the "Bacillus of Weeks"; (2) sub-acute conjunctivitis, caused by a diplo-bacillus; (3) gonorrhœal conjunctivitis. In two of these he says that protargol is most efficacious (used in 5 per cent. solution, dropped into the eye several times a day), viz., the "acute contagious" and "gonorrhœal," but that in the inflammation caused by the diplobacillus improvement is effected with protargol just as it is by nitrate of silver, but is only limited, and sulphate of zinc must be resorted to to complete the cure.

**"Cassaripe" in Infectious Eye Diseases.**—S. D. Risley[4] recommends "Cassaripe" in the treatment of corneal ulcers and infectious diseases of the eye. He has used it in a 10 per cent. ointment (as has Chandler, of Boston, who first introduced it), but says, "as it causes no irritation I do not see why the ointment should not be stronger." The ointment was applied freely between the lids and the eye subjected to massage to thoroughly distribute it; this treatment was carried out three times a day. In a few minutes after the application, in new cases, the discomfort was much diminished and the improvement rapid in comparison with other forms of treatment. In ophthalmia neonatorum the eye was well cleansed, the cassaripe ointment applied, and a supply given to be used three times daily at home after the usual cleansing of the eye. In two days the purulent discharge had entirely ceased.

**Salicylic Acid in Granular Conjunctivitis.**—Mons. Moby[5] suggests the application of salicylic acid to the conjunctiva in cases of granular conjunctivitis. The solution used is one in alcohol, of a strength of one in ten, and is applied to the granular surface with a pledget of absorbent cotton wound on a holder, dipped in the solution and squeezed dry. The application only needs to be of a few seconds' duration. The treatment is somewhat painful at first, but the pain is soon over, and is followed by immediate relief, much greater than with other local applications. It appears that the cornea is not affected by the salicylic acid. The cauterisations are repeated at first daily, then each alternate day, and finally once or twice a week. No other treatment seems to be necessary in uncomplicated cases. It is said that the addition of a one per cent. solution of cocaine to the salicylic acid solution would prevent the occurrence of the pain, but we should think this doubtful.

[1] Clin. Excerpts, Dec., 1899. [2] Brit. Med. Jour., Dec. 31, 1899. [3] Med. News, Jan. 7, 1899. [4] Brit. Med. Jour., Nov. 26, 1899. [5] New York Med. Jour., Feb. 4, 1899.

